# Sex Differences in Intestinal P-Glycoprotein Expression in Wistar versus Sprague Dawley Rats

**DOI:** 10.3390/pharmaceutics14051030

**Published:** 2022-05-10

**Authors:** Christine M. Madla, Yujia Qin, Francesca K. H. Gavins, Jing Liu, Liu Dou, Mine Orlu, Sudaxshina Murdan, Yang Mai, Abdul W. Basit

**Affiliations:** 1 Department of Pharmaceutics, UCL School of Pharmacy, University College London, 29–39 Brunswick Square, London WC1N 1AX, UK; christine.madla.16@ucl.ac.uk (C.M.M.); francesca.gavins.13@ucl.ac.uk (F.K.H.G.); m.orlu@ucl.ac.uk (M.O.); s.murdan@ucl.ac.uk (S.M.); 2 School of Pharmaceutical Sciences (Shenzhen), Sun Yat-Sen University, Guangzhou 510275, China; qinyj25@mail2.sysu.edu.cn (Y.Q.); liuj663@mail2.sysu.edu.cn (J.L.); liu.dou.14@alumni.ucl.ac.uk (L.D.)

**Keywords:** animal models, sex differences, rodents, pharmacokinetics and pharmacodynamics, oral bioavailability, protein quantification, P-glycoprotein, MDR1, ABCB1, efflux transporters

## Abstract

Wistar and Sprague Dawley are the most common strains of rat used in pharmaceutical research and are used interchangeably in pre-clinical drug development. No studies have assessed whether Wistar and Sprague Dawley rats are equivalent in the gastrointestinal factors that influence oral drug absorption, specifically in relation to intestinal transporters. Enzyme-linked immunosorbent assay (ELISA) and liquid chromatography–tandem mass spectrometry (LC-MS/MS) are two reliable methods for quantifying intestinal protein levels with their own distinct advantages and limitations. In this study, P-glycoprotein (P-gp), a key efflux transporter, was quantified using ELISA and LC-MS/MS along the complete intestinal tract of male and female Wistar and Sprague Dawley rats. This work presents that Sprague Dawley rats have innately higher baseline P-gp expression than Wistar rats. Significant sex differences in P-gp expression were identified in the jejunum, ileum and colon between male and female Wistar rats using both techniques, with males exhibiting higher P-gp levels. Sprague Dawley rats showed no sex differences in P-gp expression through ELISA and LC-MS/MS. Both methods demonstrated similar trends for P-gp quantification, but ELISA could offer faster data acquisition. Our findings report significant sex differences between the strains and highlight that Wistar and Sprague Dawley rats are not equivalent in their P-gp expression. As humans exhibit distinct sex differences in intestinal P-gp levels, Wistar rats may therefore be a more suitable pre-clinical animal strain to model oral drug absorption of P-gp substrates in male and female subjects.

## 1. Introduction

Animal models are an integral pillar in pre-clinical development that have aligned our understanding of the pathologies of diseases and drug-target identification. Rodents have been historically and favourably used across pharmaceutical research to infer clinical decisions considering safety, pharmacokinetics, pharmacodynamics and biodistribution [1,2]. Rats are chosen as model organisms due to their widespread availability, low breeding costs, short reproductive cycle and, to some respect, similarities to humans [3]. The *Rattus* genus is an umbrella for multiple species of rats. The first documented rat was the wild brown *Rattus norvegicus* named by English scientist Berkenhout in 1772 who believed that these rats migrated to England from Norwegian ships in 1728 [4]. Over time, rats were bred for sport producing variants including the albino mutant *Rattus norvegicus*. In 1906, the Wistar Institute bred a strain from the albino mutant *Rattus norvegicus*, named the Wistar rat [5]. Notably, it was the first standardised rat model to be used in biological and medical research. The Sprague Dawley rat, however, is an outbred rat, first produced in 1925 from a Wistar female and a hybrid male of unknown origins in the Sprague-Dawley Animal Company. Later, Charles River developed a new line with better microbial status by caesarean derivation [6].

While other many other strains exist, Wistar and Sprague Dawley rats are the most common strains in pharmaceutical pre-clinical studies. A study by Musther et al. reviewed over 122 articles from 1969 to 2012 and found that the Sprague Dawley strain accounted for 49% of studies concerning oral and intravenous bioavailability, potentially due to their calm nature and faster rate of growth [7]. In contrast, 35% of research focused on Wistar rats. Other strains including the albino rat, Long Evans, Fisher 344 and Lewis types accounted for 16% [8]. There is an under-appreciation for the differences and nuances in innate rat physiology which could influence the outcome of drug-response research. As a consequence, little thought is invested in selecting the rat strain in pre-clinical absorption and pharmacokinetic testing. As much as inter-individual differences occur in humans, a similar view could be applied to pre-clinical models. Intestinal membrane transporters, such as clinically relevant P-glycoprotein (P-gp), are coined as ‘housekeeping’ proteins which prevent the uptake of xenobiotics into blood capillaries [9,10]; as such, they represent a natural barrier to oral drug absorption [11]. P-gp, also known as multidrug resistance protein 1 (MDR1), determines the absorption, distribution and elimination of many drug classes, which ultimately governs the plasma and tissue concentrations [9,12] of substrates including dabigatran etexilate, digoxin and fexofenadine [13]. In 2020, the Food and Drug Administration (FDA) recommended that bioactive compounds should be screened as early as possible in the drug-discovery pipeline to identify whether they are P-gp substrates [14]. Differences in P-gp mRNA levels and protein expression have been previously described in Wistar rats, although results were dependent on the quantification strategy used [15,16,17]. Specifically, sex differences were observed in the expression of P-gp in Wistar rats with protein levels being higher in males than females from the proximal to the distal small intestine. A strong positive linear correlation (*R*^2^ = 0.85) for P-gp expression was identified comparing Western blot and liquid chromatography–tandem mass spectrometry (LC-MS/MS) [18].

LC-MS/MS-based proteomics is a robust method for protein quantification with high sensitivity, although it can be subject to high costs and challenging method development and validation [19]. Immunohistochemistry techniques, such as the enzyme-linked immunosorbent assay (ELISA) method, have been used for the quantification of proteins in both in vitro and in vivo matrices. Commercially available ELISA kits have been fully validated for their limit of detection, linearities and specificity, and require limited sample preparation [20]. P-gp expression from in vitro models has been correlated to poor patient prognosis in numerous studies as levels in patient samples did not reach what was observed in cultured cells [21]. Understanding the innate differences in commonly used rat models, therefore, could aid the translation of P-gp drug products from pre-clinical to first-in-human trials [22] following accelerated protein quantification techniques.

This study aims to be the first to investigate strain and sex differences in intestinal P-gp expression between two commonly used rat models in pharmaceutical research, the Wistar and Sprague Dawley strains. Quantification methods were also assessed for their advantages in detecting potential differences to infer the most appropriate rat strain for P-gp drug substrate development for effective translation to humans.

## 2. Materials and Methods

### 2.1. Materials

Krebs-bicarbonate Ringer solution (KBR), pH 7.4, was freshly prepared before the experiment at room temperature and was kept at 37 °C. KBR was composed of 10 mM D-glucose, 1.2 mM CaCl_2_, 1.2 mM MgCl_2_, 115 mM NaCl, 25 mM NaHCO_3_, 0.4 mM KH_2_PO_4_ and 2.4 mM K_2_HPO_4_ [23]. Lysis buffer was freshly prepared with 50 mM Tris, 250 mM NaCl, 5 mM ethylenediaminetetraacetic acid (EDTA), 1 mM Na_3_VO_4_, 1 mM phenylmethylsulfonyl fluoride (PMSF), 1% Nonidet P40 and protease inhibitor cocktail from Sigma (Darmstadt, Germany) in a phosphate-buffered saline (PBS) solution and stored at 4 °C.

The surrogate peptide for P-gp quantification and its stable isotope-labelled internal standard were of analytical grade (purity >95%) and were synthesised and quantified via the amino acid analysis using an Allpeptide (Hangzhou, China). All other chemicals are mentioned individually in the following methods. Rat P-gp ELISA kits (MM-0604R2) were purchased from MEIMAN Biotech (Guangzhou, China). 

### 2.2. Animals

In total, 8 male and 8 female Wistar and Sprague Dawley rats (healthy, from 8 to 13 weeks old), respectively, were used as the animal models. The rats were housed at room temperature (25 °C) in a light–dark cycle of 12 h. The rats acclimatised to the animal unit for at least 7 days. On the day before the experiments, the rats were fasted overnight and housed individually in metabolic cages until the following morning at 8 a.m. The protocols were agreed by the Administrative Committee on Animal Research in Sun Yat-sen University (Protocol No. SYSU-20200420; date of approval: 1 January 2021). Full compliance with the Animal Research: Reporting of In Vivo Experiments (ARRIVE) guidelines was practiced and followed.

### 2.3. Intestinal Tissue Collection

Their intestines were immediately excised and stored in an ice-cold KBR solution, following sacrifice by CO_2_ asphyxiation. The intestines were cut into four segments, roughly 2 cm pieces: duodenum (1 cm from the ligament of Treitz), jejunum (10 cm from the ligament of Treitz), ileum (1 cm from the caecum) and colon (descending), were isolated and opened along their mesenteric border. The tissues were gently washed with KBR solution to remove the intestinal contents.

### 2.4. Initial Total Protein Quantification

The mucosal tissues (approximately 50 mg) were cut into small pieces and homogenized in 0.5 mL of RIPA lysis buffer at 30 Hz for 30 s with a TissueLyser (QIAGEN, Hilden, Germany); this was repeated twice at intervals of 30 s for complete homogenisation. The tissue homogenates were incubated at 4 °C for 2 h, then centrifuged at 12,000× *g* for 5 min. The total tissue protein was collected in the supernatants; its concentration was subsequently determined with a Pierce™ BCA protein assay kit (Beyotime Biotechnology, Shanghai, China) according to the manufacturer’s instructions.

### 2.5. Measurement of P-glycoprotein (P-gp) Protein Levels by Quantitative Enzyme-Linked Immunosorbent Assay (ELISA) and Quantitative Liquid Chromatography–Tandem Mass Spectrometry (LC-MS/MS)

To measure the target P-gp transporter protein level by ELISA, ELISA kits (Meimian Biotech, Guangzhou, China) were used based on the manufacturer’s description; a volume of the supernatant (from Section 2.4) containing a mass of 50 μg of total protein lysate was taken.

To measure the target P-gp transporter protein level, LC-MS/MS was utilised following a published method [18]. Briefly, a volume of the supernatant (from Section 2.4) containing a mass of 50 μg of total protein lysate was taken and made up to 200 μL with 50 mM ammonium bicarbonate buffer. Dithiothreitol, which was freshly prepared (4 μL; 20 mM), was then added and gently mixed to form the total protein solution. The solution was incubated for 20 min at 56 °C for protein denaturation to occur. After cooling, alkylation was conducted by adding 8 μL of iodoacetamide (375 mM) and was then incubated for 20 min at 37 °C in a dark environment. The precipitation step was carried out by adding 600 μL of cold methanol and 150 μL of cold chloroform (both at 4 °C) to the sample solution. The tube was inverted several times. A volume of 450 μL of cold water was added; then, the sample was immediately centrifuged at 15,000 rpm for 5 min at 4 °C. After centrifugation, the lower layer (below the suspended protein pellet) was removed before the upper layer (above the suspended protein pellet), and an additional 450 μL of cold methanol was added. The tube was inverted to wash the protein pellet. The sample was then centrifuged at 15,000 rpm for 5 min at 4 °C. Immediately after centrifugation, the supernatant was fully removed. Ammonium bicarbonate buffer (47 μL; 50 mM) was added to the precipitated protein pellet. The protein solution was then sonicated for 30 s for a maximum of three cycles until a homogeneous protein suspension was achieved. Human serum albumin was processed under the same conditions for subsequent use as a matrix in the construction of the calibration curve. Trypsin solution (5 μL; 0.5 μg/μL) was added to the re-suspended protein solution and incubated for 4 h at 37 °C. Stable isotope-labelled internal P-gp standard (5 μL; 200 fmol/μL) was added to each sample, and unlabelled peptides were spiked into the human serum albumin matrix to serve as calibration standards or quality control samples toward method validation. The digestion process was stopped by adding 3 μL of 50% formic acid in water. The final processed sample solution (60 μL) was then centrifuged at 15,000 rpm for 5 min at 4 °C, and 30 μL of the supernatant was then obtained for LC-MS/MS analysis. All sample digestion procedures were processed in Protein LoBind^®^ tubes (Eppendorf, Hamburg, Germany).

A Shimadzu LCMS-8060 system (Kyoto, Japan) coupled with an ACQUITY UPLC CSH C18 column (2.1 mm × 50 mm, 1.7 μm; Waters, MA, USA) was used for the analyses. The mobile phases were 0.1% formic acid in water and 0.1% formic acid in acetonitrile with a flow rate of 0.5 mL/min. The gradient elution procedure started with 98% solvent A for 5 min, followed by a linear gradient from 98% solvent A to 75% solvent A over 10 min; afterwards, 75% solvent A was held for 1 min, then changed to 55% solvent A for an additional 2 min. Then, solvent A was changed back to the original state (98%) and held for 7 min until the end of the analyses. In accordance with previous studies [24,25], the surrogate peptide sequence as a proxy of P-gp abundance and the three multiple reaction monitoring (MRM) transitions were employed; they are listed in Table 1. The calibration curve was established for each MRM transition and P-gp abundance was calculated from the average value of the three transitions.

The mass spectrometer was equipped with an electrospray for ionization and operated in the positive ion mode to monitor the three *m*/*z* transitions with a 300 °C source temperature, nebuliser at 45 psi, 11 L/min sheath gas flow, 500 V nozzle voltage, 20 collision energy and 7 cell accelerator voltage. All the chromatograms were assessed with LabSolutions LCMS Ver.5.6 (Shimadzu, Kyoto, Japan). Method validation is described in our previous article [18].

### 2.6. Statistical Analysis

The data were expressed as mean ± standard deviation (S.D.) and tested for normality and homoscedasticity. As the former were true, significant differences among groups were then analysed by one-way analysis of variance (ANOVA) followed by Tukey’s post hoc analysis using IBM SPSS Statistics 19 (SPSS Inc., Chicago, IL, USA). A significance value of *p* < 0.05 was used for all tests. The correlations between ELISA and LC-MS/MS for P-gp expression were assessed by Spearman’s rank coefficient.

## 3. Results

### 3.1. Intestinal P-glycoprotein Quantification in Wistar Rats

**Figure 1:**
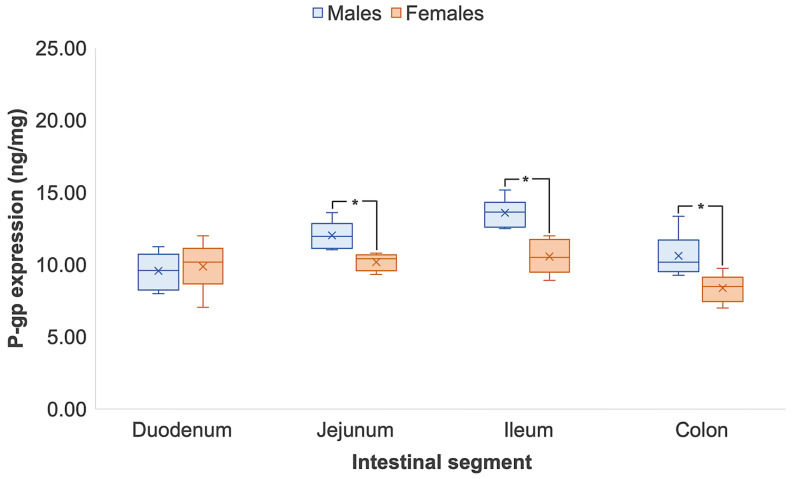
Regional and sex differences in P-gp expression in male and female Wistar rats quantified by ELISA. Data are represented as mean ± S.D., *n* = 8. The * symbol denotes statistical significance between the sexes in an intestinal region (*p* < 0.05). Regional differences are statistically significant from the small intestinal segments to the colon (*p* < 0.05).

**Figure 2:**
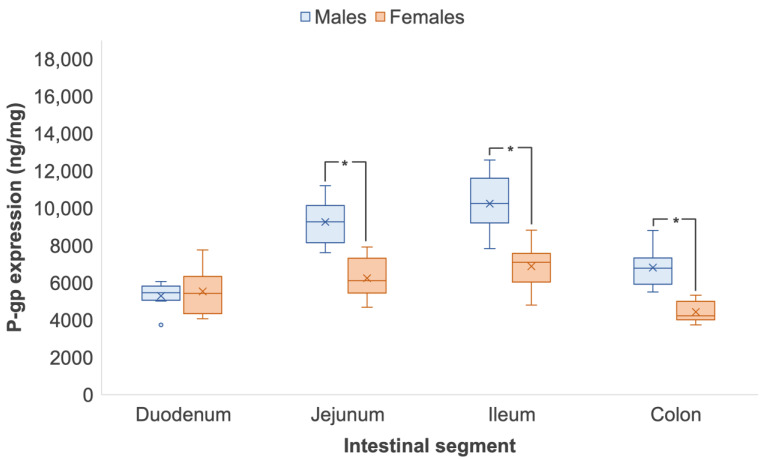
Regional and sex differences in P-gp expression in male and female Wistar rats quantified by LC-MS/MS. Data are represented as mean ± S.D., *n* = 8. The * symbol denotes statistical significance between the sexes in an intestinal region (*p* < 0.05). Regional differences are statistically significant from the small intestinal segments to the colon (*p* < 0.05).

Table 2 shows the expression of intestinal P-gp in male and female Wistar rats quantified by ELISA and LC-MS/MS (*n* = 8 respectively). The trend in male P-gp expression in both techniques was similar, whereby protein levels increased from the proximal to the distal segments of the small intestinal tract but decreased in the colon. Specifically, male P-gp expression was statistically 1.3- and 1.9-times higher in the ileum than in the duodenum, as quantified by ELISA (duodenum, 9.56 ± 1.25 ng/mg vs. ileum, 12.02 ± 0.92 ng/mg) and LC-MS/MS (duodenum, 5307.20 ± 720.63 ng/mg vs. ileum, 10,258.72 ± 1525.14 ng/mg), respectively (*p* < 0.05). Female P-gp expression, however, was consistent across the small intestine when quantified by ELISA, ranging from 9.86 to 10.56 ng/mg and presenting no statistical differences (*p* > 0.05) (Figure 1). By LC-MS/MS, P-gp expression significantly increased from the duodenum to the ileum by LC-MS/MS (*p* < 0.05) (Figure 2). At the distal segments, P-gp expression significantly dropped in both male and female Wistar rats from the ileum to the colon. By ELISA, colonic P-gp decreased by 22% in males (*p* < 0.05) and 21% in females from the ileum (*p* < 0.05). The decrease in colonic P-gp expression was more pronounced with LC-MS/MS, as protein levels reduced by 33% and 36% in males and females, respectively (*p* < 0.05) (Figure 2). Sex differences were detected in the same intestinal regions by ELISA and LC-MS/MS with P-gp expression differing in male and female Wistar rats in the jejunum, ileum and colon (*p* < 0.05). Figures S1 and S2 show good correlation of P-gp expression when quantified by ELISA and LC-MS/MS in male (*R*^2^ = 0.9726) and female (*R*^2^ = 0.9761) Wistar rats, respectively.

### 3.2. Intestinal P-glycoprotein Quantification in Sprague Dawley Rats

**Figure 3:**
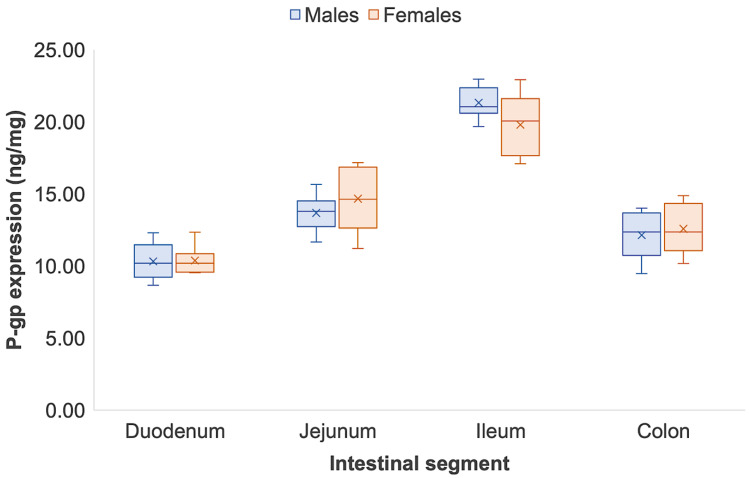
Regional and sex differences in P-gp expression in male and female Sprague Dawley rats quantified by ELISA. Data are represented as mean ± S.D., *n* = 8. Regional differences are statistically significant from the small intestinal segments to the colon (*p* < 0.05).

**Figure 4:**
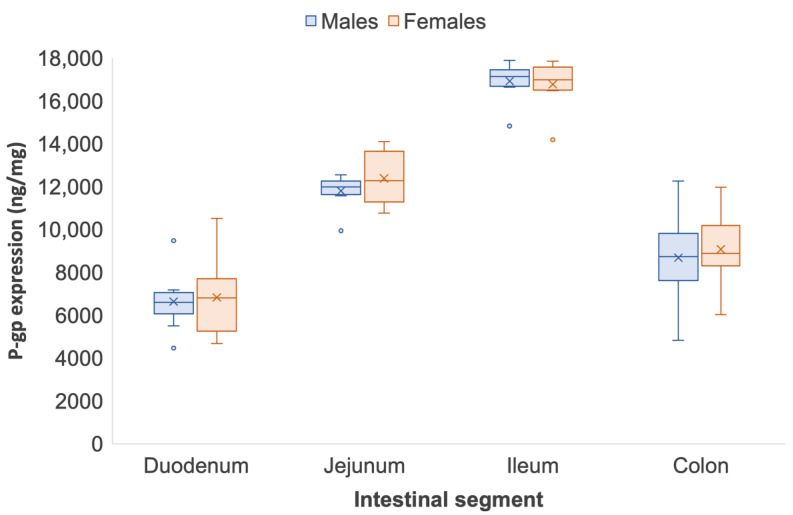
Regional and sex differences in P-gp expression in male and female Sprague Dawley rats quantified by LC-MS/MS. Data are represented as mean ± S.D., *n* = 8. Regional differences are statistically significant from the small intestinal segments to the colon (*p* < 0.05).

The intestinal P-gp expression in male and female Sprague Dawley rats quantified by ELISA and LC-MS/MS is shown in Table 3. As reported in the Wistar rat, the trend in P-gp expression across the complete intestinal tract in Sprague Dawley rats was similar between ELISA and LC-MS/MS quantification. Figure 3 and Figure 4 show that P-gp expression in Sprague Dawley rats increased along the small intestine and experienced a sharp reduction in the colon. Detected by ELISA, P-gp expression from the duodenum to the ileum increased by 2.0 (duodenum, 10.33 ± 1.25 ng/mg vs. ileum, 21.33 ± 1.10 ng/mg) and 1.9 times (duodenum, 10.38 ± 0.94 ng/mg vs. ileum, 19.80 ± 2.19 ng/mg) in males and females, respectively (*p* < 0.05) (Figure 3). Regional differences across the small intestine in male and female Sprague Dawley rats, however, were more pronounced when quantified by LC-MS/MS. From the duodenum to the ileum, P-gp significantly increased by 2.5 times (duodenum, 6633.03 ± 1452.23 ng/mg vs. ileum, 16,314.93 ± 2042.77 ng/mg) in males and 2.4 times (duodenum, 6825.23 ± 1860.21 ng/mg vs. ileum, 16,136.16 ± 2071.22 ng/mg) females, respectively (*p* < 0.05) (Figure 4). Similar to Wistar rats, a significant reduction in P-gp expression was identified from the small intestine to the colon. Quantification via ELISA showed that in males and females, colonic P-gp expression reduced by 43% and 36% from the ileum, respectively (*p* < 0.05). From the ileum, colonic P-gp significantly decreased by 49% and 46% in both male and female Sprague Dawley rats when quantified by LC-MS/MS (*p* < 0.05). Neither the ELISA nor the LC-MS/MS quantification strategy identified any sex differences in P-gp expression across the segments of the intestinal tract of Sprague Dawley rats (Figure 4). Figures S3 and S4 show good correlation of P-gp expression when quantified by ELISA and LC-MS/MS in male (*R*^2^ = 0.9862) and female (*R*^2^ = 0.9760) Sprague Dawley rats, respectively.

### 3.3. Strain Differences in Intestinal P-glycoprotein between Wistar and Sprague Dawley Rats

There were distinct differences in P-gp expression between the Wistar and Sprague Dawley rats (Figures S5–S8). Both ELISA and LC-MS/MS confirmed that P-gp expression was innately higher Sprague Dawley rats in comparison to Wistar types. Specifically, P-gp in the male and female jejunum and ileum, and in the female colon was higher in Sprague Dawley than Wistar rats by LC-MS/MS (*p* < 0.05) (Table 2 and Table 3). The quantification of protein expression via ELISA, however, was not able to detect strain differences between male Wistar and Sprague Dawley rats (*p* > 0.05).

## 4. Discussion

Wistar and Sprague Dawley rats are the most routinely used strains in pre-clinical drug-absorption studies [8,26]. A notable commentary by Clause, however, stated that by its uniformity, quality, efficiency of production and application to scientific practice, the Wistar rat is the right choice toward a standardised animal model for translation to humans [5]. 

Researchers select the rat strain for their experiments based on biological features, convention, or even cost. There are, however, key differences in their behaviour, appearance and, importantly, innate physiology. For example, Sprague Dawley rats grow faster than Wistar rats. Wistar rats, on the other hand, show higher survival rates [27]. A study by Rybnikova et al. demonstrated that Sprague Dawley rat strains exhibited higher testosterone levels in comparison to Wistar rats, contributing to their higher reproductive potential [28]. Sprague Dawley rats also presented increased motor activity and explorative levels in comparison to the Wistar rat; however, grooming patterns and depressive-like behaviours were higher in the latter [28]. 

Our study identified that Sprague Dawley rats had intrinsically higher expression of P-gp (Figure 3 and Figure 4) than Wistar rats (Figure 1 and Figure 2) following quantification by both ELISA and LC-MS/MS. A study by Oltra-Noguera et al., however, found that the mean permeability (P_eff_) of verapamil, a known P-gp substrate, was higher in Sprague Dawley rats than in Wistar rats in an in situ closed-loop perfusion model, which implies higher P-gp expression in Wistar rats [29]. On the other hand, a study by Liu et al., reported that P-gp-mediated efflux was the limiting factor for intestinal absorption of SNX-2112, a promising anti-cancer agent, in intestinal microsomes from male Sprague Dawley rats [30]. 

Biological sex can contribute towards differences in physiology, which can in turn influence drug response [31]. In pre-clinical studies, male animals can be preferentially selected over female animals due to the lack of hormonal fluctuations, ease in handling and faster clearance [32,33]. In some cases, male and female animals are pooled together to reduce the number of animals used in line with the 3Rs initiative [34]. However, these strategies ignore sex-related differences in drug performance, with implications for the clinical safety and efficacy of pharmaceutical formulations. For example, the oral bioavailability of P-gp drug substrate ranitidine is markedly higher in male rats when co-formulated with polyoxyethylated excipients, including polyethylene glycol (PEG) 400 [35,36], PEG 2000, Cremophor RH 40, Poloxamer 188 and Tween 80. No differences in ranitidine oral bioavailability, however, was seen in female rats [37]. The implications of PEG 400 were demonstrated to have clinical consequences in humans. Male volunteers showed statistically higher oral bioavailability of the P-gp drug substrates ranitidine [38] and cimetidine [39] than the female counterparts due to the excipient modulating baseline P-gp expression in males alone.

Our results demonstrated distinct differences between the two rat strains, with respect to sex as a biological variable. Small intestinal P-gp in male Wistar rats were statistically higher than their female counterparts when quantified by both ELISA and LC-MS/MS. In contrast, there were no sex differences in P-gp expression seen in Sprague Dawley rats. Oltra-Noguera et al. reported a significant sex difference in the intestinal permeation of verapamil in Wistar rats but not Sprague Dawley rats [29], which corroborates the findings of this study. Evidence of lower P-gp activity in female Wistar rats has been demonstrated by Mariana et al. Rhodamine 123, a well-known P-gp substrate, showed 1.78-fold greater serosal-to-mucosal transport in males than females, which suggests increased P-gp activity [40]. Using a closed-loop model, Yin et al. showed reduced intestinal absorption of P-gp substrate ampicillin in female Wistar rats in comparison with the male counterparts [41].

The optimal site for oral drug absorption in the gastrointestinal tract is dependent on the properties of the drug products and regional differences in the mucus layer, intercellular tight junctions, surface area and the expression of transporters and enzymes in the intestinal epithelial cells [42]. The trend in the P-gp level across the small intestine reflects our groups’ previous findings in Western blot and LC-MS/MS, where P-gp increases from the proximal to the distal small intestine and then decreases in the colon [18]. 

A reliable and fast analytical method is required to support clinical drug development. LC-MS/MS and immunosorbent assays are often utilised to aid the understanding of the effect of pharmaceutical formulations. One application is the quantification of P-gp drug expression to infer for potential interactions or inhibition [18,43,44]. Protein precipitation, conventional liquid/liquid extraction and solid-phase extraction procedures are routine stages of sample preparation for LC-MS/MS. Most of these preparation techniques take at least 1 hour to perform, without the consideration of incubation periods [45]. Although solid phase extraction is relatively simple, there is a drying process to ensure the separation of the protein pellet. This involves the extraction of the solvent; however, if the solvent is not completely removed, issues with reversed-phase liquid chromatography compatibility may arise [46]. Slow and technically difficult sample preparation and long sample cycle times can therefore be a limiting factor in analyses and drug-development decision making. With the demand of fast-paced development and improved toxicological and efficacy understanding for new modalities, high-throughput strategies are being adopted more and more in pharmaceutical research [43]. The 96-well plate is the most used format for bioanalytical sample preparation. Singleplex ELISA assays have been designed to be highly sensitive and quantitative for the detection of target analytes within heterogenous samples [44], with quantification times reduced by at least half in comparison to LC-MS/MS [47]. The work herein identified that the trend in P-gp expression between the sexes and between Wistar and Sprague Dawley rats along the intestinal tract were similar when quantified by ELISA and LC-MS/MS. Sex differences in small intestinal and colonic P-gp levels between ELISA and LC-MS/MS were also appreciated. The significant increase in P-gp expression in female Wistar rats, however, was detected only by LC-MS/MS, potentially due to a wider assay range or immunoaffinity [48]. This finding was less pronounced through the immunosorbent assay method, which may have been due reduced detection sensitivity, restrictive analytical range [49] or the small sample size (*n* = 8) (Figure 1 and Figure 2). Although both techniques showed similar trends of intestinal expression and displayed good positive correlation (*R*^2^ = 0.9726 – 0.9862; Supplementary Figures S1–S4), LC-MS/MS measurements were markedly higher than ELISA measurements due to superior specificity to total protein quantification and invariance to cross-reactions, in comparison to the ELISA method which measures the bound target protein [50,51].

As P-gp expression is higher in Sprague Dawley rats, should this strain be the pre-clinical animal model of choice for a new modality that is investigated to be a P-gp substrate? Drug response may present decreased oral bioavailability from greater P-gp efflux in comparison to Wistar rats. Higher expression of P-gp may be linked to greater protection from xenobiotics entering systemic circulation [52]. However, the sex differences in P-gp expression as shown in humans [18] are not exhibited in Sprague Dawley rats. Drug development has taken a ‘one size fits all’ approach towards pharmaceutical manufacturing. The adoption of sex-informed perspectives across the pharmaceutical pipeline, however, increases scientific rigour and encourages novel research [53]. This inclusion in pre-clinical development, therefore, can expedite translation into first-in-human trials if sex is considered as a biological variable in animal studies. Wistar rats demonstrate key sex differences in innate P-gp expression in the small intestine where the majority of immediate release drugs are absorbed. As such, this study advocates for this animal model to be used when investigating potential P-gp drug substrates, so that sex-related influences are powered in research and development [31,54].

## 5. Conclusions

This study is the first to identify strain differences in the intestinal P-gp expression between Wistar and Sprague Dawley rats. Wistar rats showed significant sex differences in the P-gp expression along the intestinal tract—duodenum, jejunum, ileum and colon. Sprague Dawley rats, however, presented no sex differences in intestinal P-gp levels. Importantly, both quantification strategies—ELISA and LC-MS/MS—produced similar trends in results concerning intestinal P-gp expression. Although LC-MS/MS may be more sensitive, ELISA can lead to faster data acquisition to effectively guide the next stages of drug development. This study proposes that Wistar rats should be the animal model of choice for pre-clinical investigations concerning the oral development of P-gp drug substrates due to the appreciation of innate sex differences as seen in humans.

## Figures and Tables

**Table 1:** Tryptic proteospecific peptide and its respective ions and mass transitions used for P-glycoprotein (P-gp) quantification.

Molecule	Peptide Sequence	Mass	Transition Number	Q1 *m*/*z*	Q1-CE	Q3 *m*/*z*	Q3-CE
ABCB1 (P-gp)	AGAVAEEVLAAIR (surrogate peptide)	1268.7	1	635.3	30	771.3	30
			2	635.3	30	900.5	30
			3	635.3	30	971.6	30
	AGAVAEEVLAAIR ^a^ (internal standard)	1278.6	1	640.3	30	781.4	30
			2	640.3	30	910.5	30
			3	640.3	30	981.5	30

^a^ Isotope-labelled amino acid; the labelling of Arg (R) was conducted by introducing C^13^ and N^15^.

**Table 2:** P-glycoprotein expression in male and female Wistar rats quantified by enzyme-linked immunosorbent assay (ELISA) and liquid chromatography–tandem mass spectrometry (LC-MS/MS).

Intestinal P-gp in Male Wistar Rats										
Quantified by ELISA (ng/mg) (*n* = 8)										
Intestinal segments	1	2	3	4	5	6	7	8	Mean	S.D.
Duodenum	10.78	7.98	8.08	10.55	9.27	9.91	11.23	8.71	9.56	1.25
Jejunum	12.59	11.03	11.03	12.07	12.13	11.43	11.83	13.08	12.02	0.92
Ileum	12.57	12.51	13.95	14.12	14.38	12.67	13.33	15.16	13.59	0.97
Colon	12.06	9.27	10.52	9.45	13.35	9.77	9.82	10.67	10.61	1.42
Quantified by LC-MS/MS (ng/mg) (*n* = 8)										
Intestinal segments	1	2	3	4	5	6	7	8	Mean	S.D.
Duodenum	5894.88	3746.03	5030.55	5634.86	5152.56	6074.69	5399.90	5524.15	5307.20	720.63
Jejunum	11,206.95	7938.44	7624.05	8952.71	9580.88	8787.68	9770.73	10,273.60	9266.88	1190.55
Ileum	7838.09	9156.47	10,342.37	12,591.57	9395.06	10,494.22	11,978.40	10,176.80	10,258.72	1525.14
Colon	7447.57	5741.64	6694.71	5513.41	6896.77	6474.12	8805.73	6987.12	6820.13	1026.52
**Intestinal P-gp in Female Wistar Rats**										
Quantified by ELISA (ng/mg) (*n* = 8)										
Intestinal segments	1	2	3	4	5	6	7	8	Mean	S.D.
Duodenum	10.52	11.32	8.45	9.27	11.99	10.37	9.99	7.04	9.87	1.59
Jejunum	10.80	10.61	9.57	9.57	10.31	10.70	10.49	9.33	10.17	0.59
Ileum	8.91	10.87	9.28	12.00	10.04	11.42	10.16	11.85	10.57	1.16
Colon	7.90	7.01	8.91	9.74	8.94	9.19	7.29	8.09	8.39	0.96
Quantified by LC-MS/MS (ng/mg) (*n* = 8)										
Intestinal segments	1	2	3	4	5	6	7	8	Mean	S.D.
Duodenum	5280.92	7759.25	4080.69	4081.68	5595.60	5900.99	6495.27	5138.45	5541.61	1222.15
Jejunum	5953.21	6276.65	5625.97	4700.24	7927.81	7582.72	6552.90	5402.36	6252.73	1087.68
Ileum	4810.97	6245.36	5978.92	8828.72	7429.65	7048.28	7631.05	7159.52	6672.04	1310.89
Colon	4131.86	3975.40	5079.73	4132.10	5341.54	4783.79	3752.69	4351.89	4443.62	563.90

**Table 3:** P-glycoprotein expression in male and female Sprague Dawley rats quantified by enzyme-linked immunosorbent assay (ELISA) and liquid chromatography–tandem Mass spectrometry (LC-MS/MS).

Intestinal P-gp in Male Sprague Dawley Rats										
Quantified by ELISA (ng/mg) (*n* = 8)										
Intestinal segments	1	2	3	4	5	6	7	8	Mean	S.D.
Duodenum	10.13	8.67	12.32	9.09	9.68	10.71	10.28	11.75	10.33	1.25
Jejunum	14.38	12.67	15.67	12.97	14.18	14.60	13.43	11.67	13.69	1.26
Ileum	21.07	19.67	20.58	20.71	22.43	21.03	22.97	22.19	21.33	1.10
Colon	9.49	10.66	13.51	14.02	12.21	11.02	12.52	13.75	12.15	1.63
Quantified by LC-MS/MS (ng/mg) (*n* = 8)										
Intestinal segments	1	2	3	4	5	6	7	8	Mean	S.D.
Duodenum	5498.43	4463.30	9478.77	6244.87	7007.73	6393.02	6805.60	7172.54	6633.03	1452.23
Jejunum	12,154.04	9940.48	12,551.53	11,652.34	11,578.94	12,553.57	12,129.70	11,834.41	11,799.37	836.35
Ileum	17,073.66	14,931.03	17,894.74	16,642.60	17,684.77	17,182.61	17,375.64	16,694.31	16,314.93	2042.77
Colon	4821.69	6249.81	11,198.55	12,258.88	9356.98	8422.04	8074.38	8553.51	8679.00	2419.33
**Intestinal P-gp in Female Sprague Dawley Rats**										
Quantified by ELISA (ng/mg) (*n* = 8)										
Intestinal segments	1	2	3	4	5	6	7	8	Mean	S.D.
Duodenum	12.35	10.12	9.66	9.54	10.93	10.26	10.65	9.56	10.38	0.94
Jejunum	15.20	14.07	16.51	11.23	16.98	14.01	17.19	12.20	14.67	2.21
Ileum	21.12	19.17	17.63	17.09	17.78	22.94	20.97	21.78	19.81	2.19
Colon	12.08	10.84	14.53	11.74	10.18	13.78	12.64	14.89	12.59	1.71
Quantified by LC-MS/MS (ng/mg) (*n* = 8)										
Intestinal segments	1	2	3	4	5	6	7	8	Mean	S.D.
Duodenum	10,509.45	6445.07	5168.71	4678.34	5483.31	7807.17	7162.20	7347.60	6825.23	1860.21
Jejunum	12,708.23	11,088.31	14,104.71	12,248.01	12,305.06	13,957.70	10,766.36	11,868.85	12,380.90	1202.86
Ileum	17,848.27	17,492.35	14,189.24	16,474.53	16,597.27	17,608.26	17,010.48	16,952.30	16,136.16	2071.22
Colon	8845.54	6019.95	11,973.16	8319.22	9703.36	10,345.87	8920.06	8553.51	9072.58	1724.76

## Supplementary Materials

The following supporting information can be downloaded at: https://www.mdpi.com/article/10.3390/pharmaceutics14051030/s1, Figure S1: Correlation of intestinal P-gp expression when quantified by ELISA and LC-MS/MS in male Wistar rats, Figure S2: Correlation of intestinal P-gp expression when quantified by ELISA and LC-MS/MS in female Wistar rats, Figure S3: Correlation of intestinal P-gp expression when quantified by ELISA and LC-MS/MS in male Sprague Dawley rats, Figure S4: Correlation of intestinal P-gp expression when quantified by ELISA and LC-MS/MS in female Sprague Dawley rats, Figure S5: Strain differences in P-gp expression in male Wistar and Sprague Dawley rats quantified by ELISA. Data are represented as mean ± S.D., *n* = 8. The symbol // denotes statistical significance between the two strains in an intestinal region (*p* < 0.05), Figure S6: Strain differences in P-gp expression in female Wistar and Sprague Dawley rats quantified by ELISA. Data are represented as mean ± S.D., *n* = 8. The symbol // denotes statistical significance between the two strains in an intestinal region (*p* < 0.05)., Figure S7: Strain differences in P-gp expression in male Wistar and Sprague Dawley rats quantified by LC-MS/MS. Data are represented as mean ± S.D., *n* = 8. The symbol // denotes statistical significance between the two strains in an intestinal region (*p* < 0.05)., Figure S8: Strain differences in P-gp expression in female Wistar and Sprague Dawley rats quantified by LC-MS/MS. Data are represented as mean ± S.D., *n* = 8. The symbol // denotes statistical significance between the two strains in an intestinal region (*p* < 0.05).
