# Supplementary material for: Sex Differences in Intestinal P-Glycoprotein Expression in Wistar versus Sprague Dawley Rats
Source: Pharmaceutics. 2022 May 10;14(5):1030. doi: 10.3390/pharmaceutics14051030 (PMC9143158; doi:10.3390/pharmaceutics14051030)
Supplement: Supplementary file 1 [file pharmaceutics-14-01030-s001.zip › pharmaceutics-1691956-supplementary.pdf]

pharmaceutics

MDPI

Supplementary Materials: Sex Differences in Intestinal P-glycoprotein Expression in Wistar Versus Sprague Dawley Rats

Christine M. Madla, Yujia Qin, Francesca K. H. Gavins, Jing Liu [2], Liu Dou, Mine Orlu, Sudaxshina Murdan, Yang Mai and Abdul W. Basit

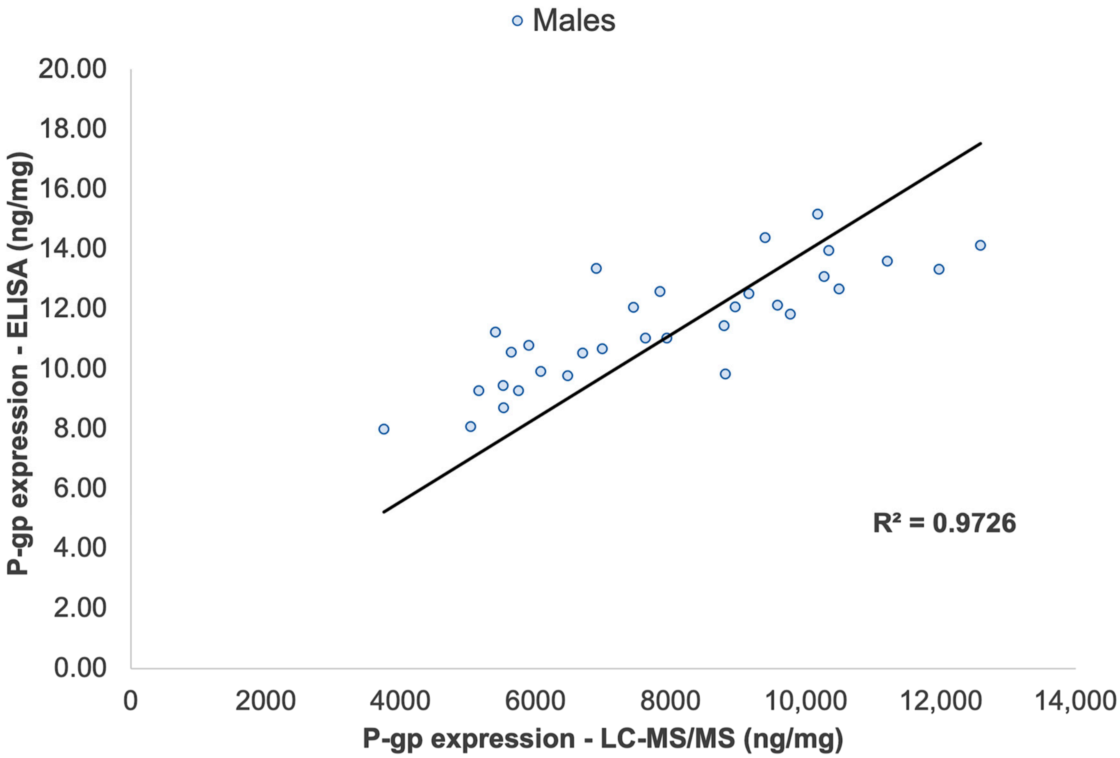

Figure S1. Correlation of intestinal P-gp expression when quantified by ELISA and LC-MS/MS in male Wistar rats.

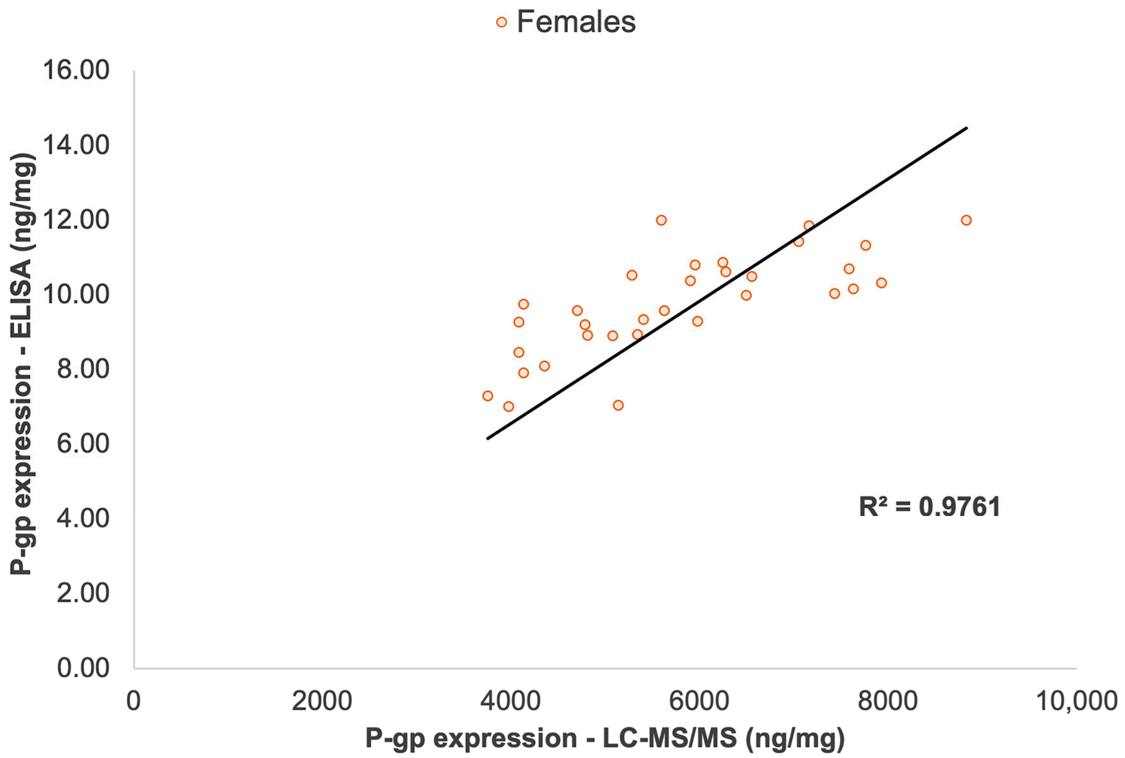

Figure S2. Correlation of intestinal P-gp expression when quantified by ELISA and LC-MS/MS in female Wistar rats.

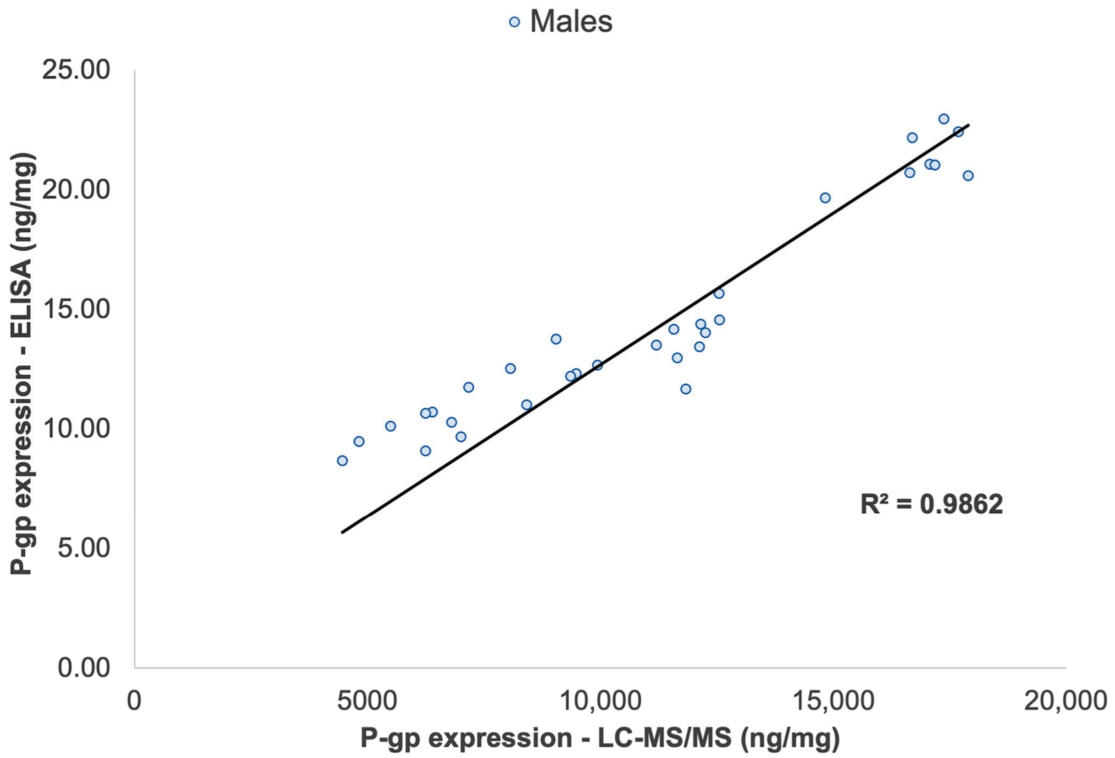

Figure S3. Correlation of intestinal P-gp expression when quantified by ELISA and LC-MS/MS in male Sprague Dawley rats.

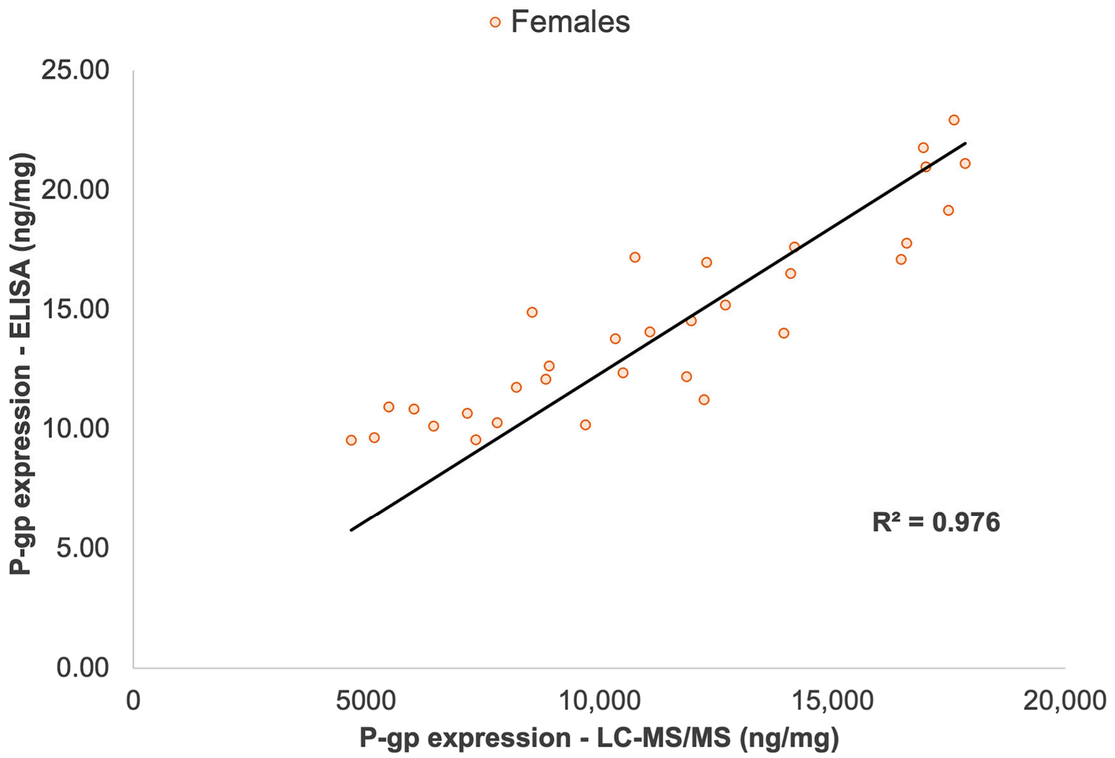

Figure S4. Correlation of intestinal P-gp expression when quantified by ELISA and LC-MS/MS in female Sprague Dawley rats.

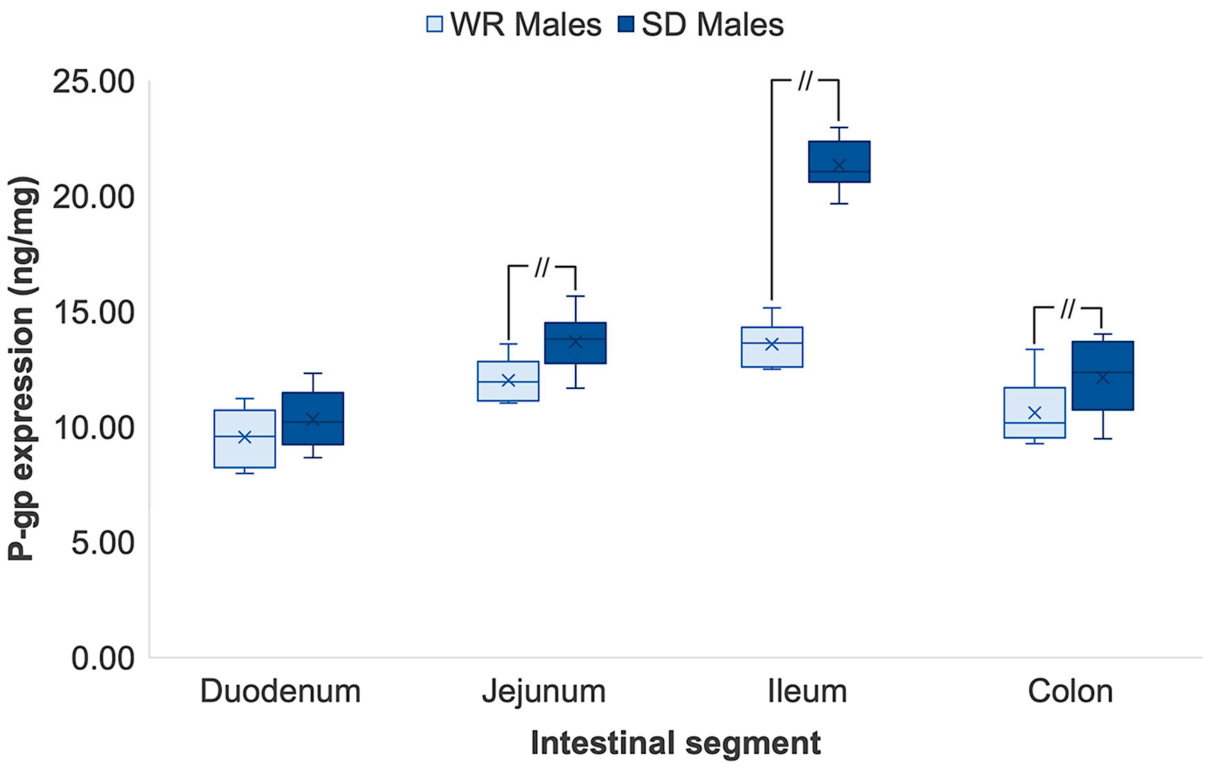

Figure S5. Strain differences in P-gp expression in male Wistar and Sprague Dawley rats quantified by ELISA. Data is represented as mean ± S.D., $n = 8$. The symbol // denotes statistical significance between the two strains at an intestinal region ($p < 0.05$).

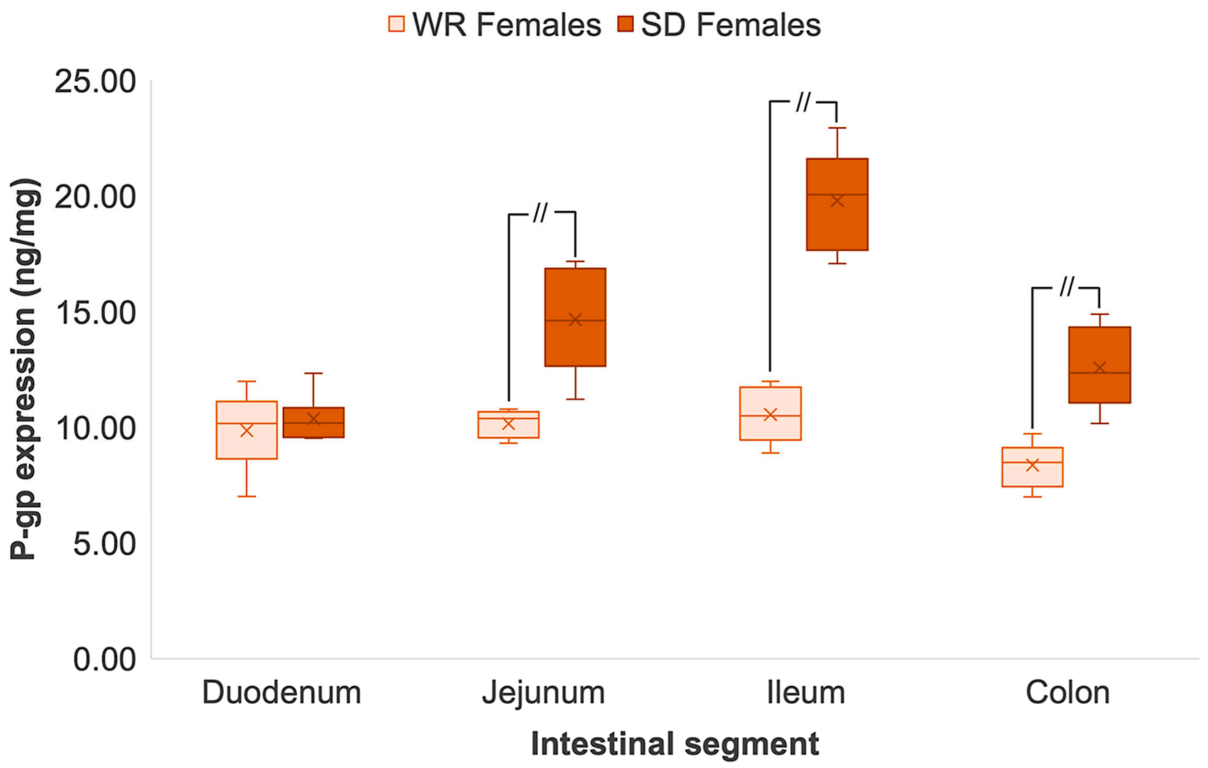

Figure S6. Strain differences in P-gp expression in female Wistar and Sprague Dawley rats quantified by ELISA. Data is represented as mean ± S.D., $n = 8$. The symbol // denotes statistical significance between the two strains at an intestinal region ($p < 0.05$).

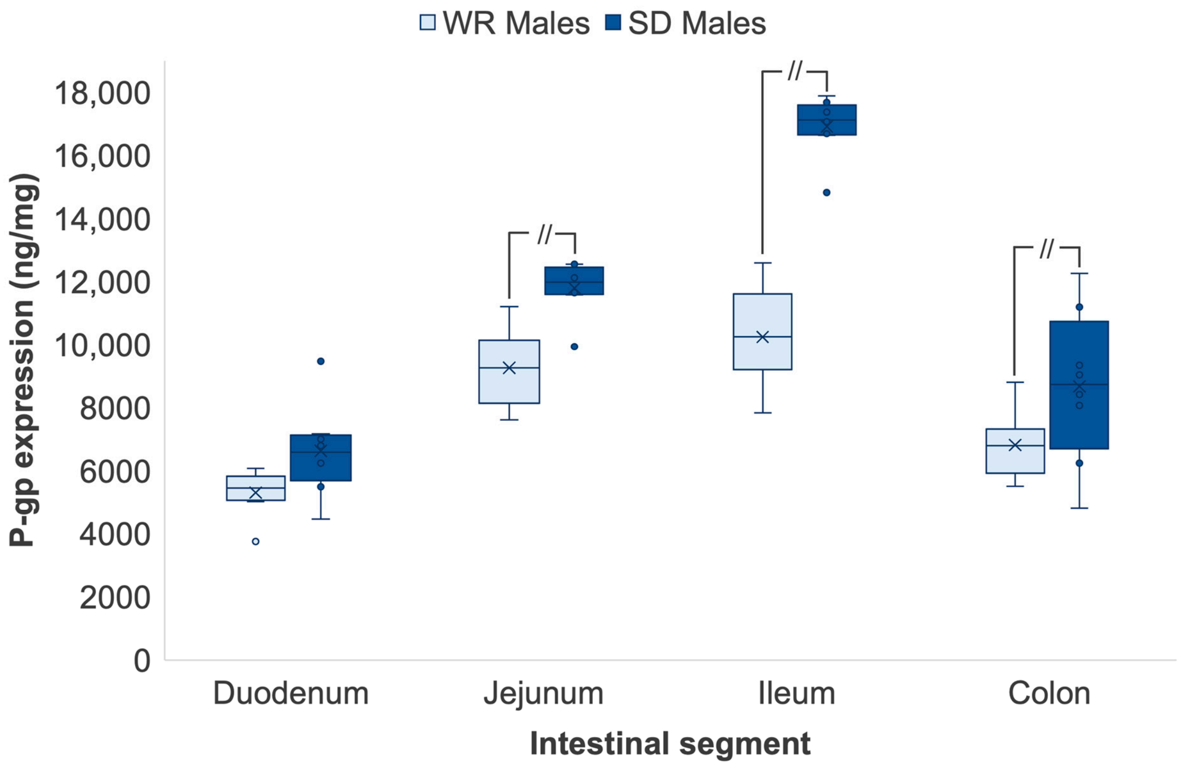

Figure S7. Strain differences in P-gp expression in male Wistar and Sprague Dawley rats quantified by LC-MS/MS. Data is represented as mean ± S.D., $n = 8$. The symbol // denotes statistical significance between the two strains at an intestinal region ($p < 0.05$).

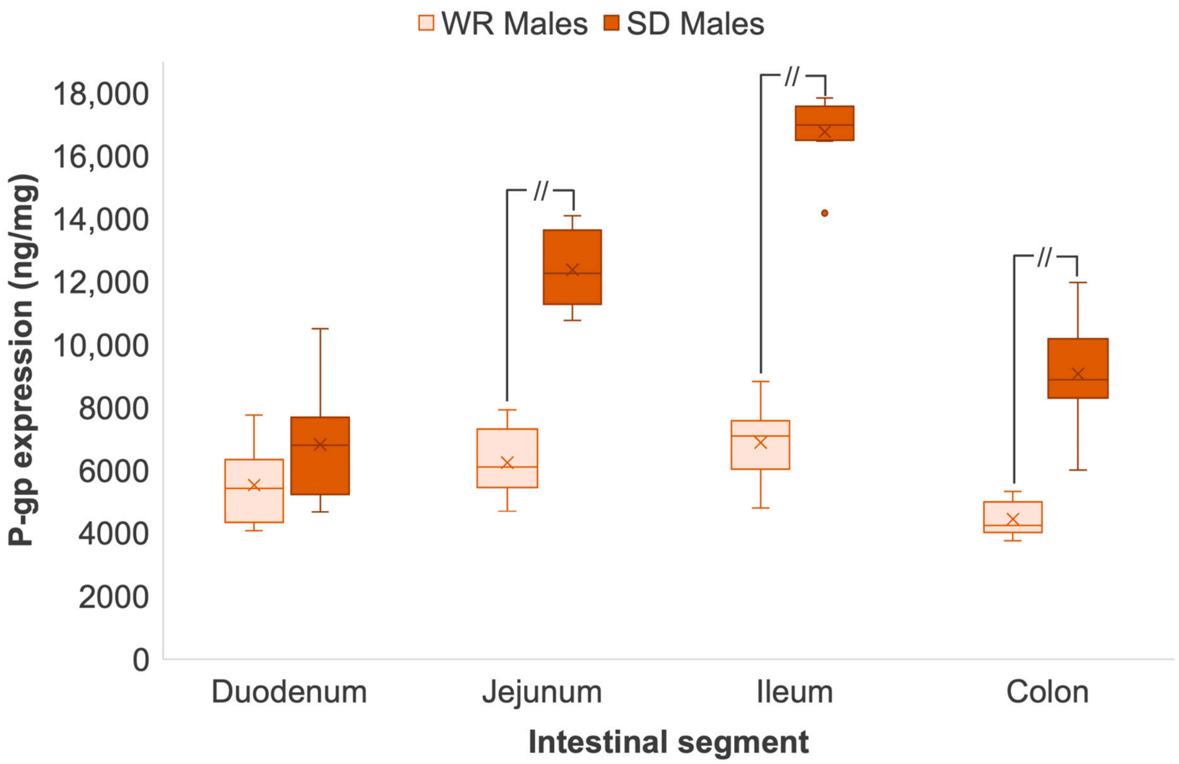

Figure S8. Strain differences in P-gp expression in female Wistar and Sprague Dawley rats quantified by LC-MS/MS. Data is represented as mean ± S.D., $n = 8$. The symbol // denotes statistical significance between the two strains at an intestinal region ($p < 0.05$).
